# Post-malaria neurological syndrome: four cases, review of the literature and clarification of the nosological framework

**DOI:** 10.1186/s12936-018-2542-8

**Published:** 2018-10-26

**Authors:** Yanis Tamzali, Sophie Demeret, Elie Haddad, Hélène Guillot, Eric Caumes, Stéphane Jauréguiberry

**Affiliations:** 1 Infectious and Tropical Diseases Unit, APHP, Pitié Salpêtrière Hospital, 75013 Paris, France; 2 Neurology Department, APHP, Pitié Salpêtrière Hospital, 75013 Paris, France; 3 Sorbonne University, INSERM, Pierre Louis Institute of Epidemiology and Public Health (UMRS 1136), Paris, France; 4 National Reference Centre for Malaria, Paris, France

## Abstract

**Background:**

Post-malaria neurological syndrome (PMNS) is a debated entity, defined by neurological complications following a post-malaria symptom-free period and a negative blood smear. Four cases of PMNS are hereby reported and a review the literature performed to clarify the nosological framework of this syndrome.

**Methods:**

A French teaching hospital infectious diseases database was investigated for all PMNS cases occurring between 1999 and 2016 and the PubMed database for cases reported by other institutions after 1997. A case was defined by the de novo appearance of neurological signs following a post-malaria symptom-free period, a negative blood smear, and no bacterial or viral differential diagnoses.

**Results:**

Four patients from the database and 48 from PubMed, including 4 following *Plasmodium vivax* infection were found matching the definition. In the institution, the estimated PMNS incidence rate was 1.7 per 1000 malaria cases overall. Of the 52 patients (mean age 33 years), 65% were men. Malaria was severe in 85% of cases, showed neurological involvement in 53%, and treated with quinine in 60%, mefloquine in 46%, artemisinin derivatives in 41%, antifolic drugs in 30%, doxycycline in 8% and other types in 8%. The mean symptom-free period was 15 days. PMNS signs were confusion (72%), fever (46%), seizures (35%), cerebellar impairment (28%), psychosis (26%), and motor disorders (13%). Cerebrospinal fluid analyses showed high protein levels in 77% (mean 1.88 g/L) and lymphocytic meningitis in 59.5% (mean 48 WBC/mm^3^) of cases. Electroencephalograms were pathological in 93% (14/15) of cases, and brain MRIs showed abnormalities in 43% (9/21) of cases with white matter involvement in 100%. Fourteen patients were treated with steroids. The 18 patients with follow-up data showed no *sequelae*. The mean time to recovery was 17.4 days.

**Conclusion:**

PMNS is a rare entity englobing neurological signs after severe or non-severe malaria. It appears after a symptom-free period. PMNS occurred following treatment of malaria with a wide range of anti-malarials. The disease is self-limiting and associated with good outcome. MRI patterns underline a possible link with acute disseminated encephalomyelitis (ADEM) or auto-immune encephalitis. *Plasmodium falciparum* and *Plasmodium vivax* should be added to the list of pathogens causing ADEM.

## Background

Falciparum malaria remains a common cause of morbidity and mortality, with an estimated 212 million cases and 429,000 deaths in 2015 [1]. The disease causes neurological impairment during its acute phase and cerebral malaria can provoke neurological *sequelae*. Additionally, a return of neurological signs after cure has been reported since 1987 [2].

A work by Senanayake et al. [3] published in 1994 may be the first large report on this phenomenon. In it, the authors described 74 Sri Lankan patients, who, during a large epidemic in the 1980s, presented an isolated, self-limiting, delayed cerebellar ataxia (DCA) syndrome 3–41 days after the onset of fever due to falciparum malaria. However, it was difficult to establish a clear relationship with a post-malaria phenomenon as the onset of neurological signs occurred while almost half (34/74) of the patients still had positive blood smears, and 11 of them had not received any anti-malarial treatment.

In 1997, Nguyen et al. [4] provided a first definition of post-malaria neurological syndrome (PMNS) in a report on 22 Vietnamese patients who presented encephalitic signs after a symptom-free period (median time of 96 h) after malaria cure, and additionally had negative blood smears for *Plasmodium falciparum* and negative etiological investigations. In their study, the use of mefloquine for severe malaria was associated with PMNS (relative risk 7.4). Since then, a number of case reports and series have been published but a clear definition and pathophysiological hypotheses for this syndrome are still lacking. PMNS may be a part of acute disseminated encephalomyelitis (ADEM) or acute post-infectious encephalitis but this too remains controversial. Finally, the medical community knows little about the condition’s underlying pathophysiology, the duration of its symptom-free period, or its outcome and prognosis, and furthermore has little in the way of diagnostic tools or treatment options. Four new cases of PMNS are herein reported and the characteristics of the cases reported since 1997 discussed further with the goal of contributing to a better understanding of this rare entity.

## Methods

### Case definition of malaria

Malaria was defined as the association of compatible clinical signs with a positive blood smear and/or antigens for a *Plasmodium* spp. The disease of patients with imported malaria in France were classified as severe or non-severe using the French 2007 classification [5].

### Case definition of PMNS

PMNS was defined as the occurrence of de novo neurological signs after a symptom-free period following acute malaria (whatever the *Plasmodium* species, i.e., *P*. *falciparum Plasmodium vivax*, *Plasmodium ovale*, *Plasmodium malariae* or *Plasmodium knowlesi*), associated with a negative blood smear and no retainable differential diagnosis. The symptom-free period was a crucial criterion.

### Case origin

All the cases fitting the case definition and seen in the department between 1999 and 2016 were included. PubMed was also investigated for neurological conditions following malaria using the following Mesh terms date-limited to ≥ 1997: “post-malaria neurological syndrome”, “post-malaria and ADEM”, “*Plasmodium*/*falciparum*/*vivax*/*ovale*/*malariae*/*knowlesi*” and “neurological” and “encephalitis” and “ADEM” and “cerebellar”.

In the resulting patient population (hospital patients plus those screened from the literature), the following data was assessed: age, gender, malaria severity, malaria treatment, symptom-free period duration, fever presence, neurological signs (confusion, cerebellar and motor impairment, seizures, psychosis), C-reactive protein (CRP), cerebral spinal fluid (CSF) characteristics, electroencephalography (EEG) signs, brain magnetic resonance imaging (MRI) results, treatment and outcome when available.

### Statistical analysis

Rates and means were calculated on the available data. Continuous variables were expressed as medians with minimum–maximum values (min–max) and means with standard deviations (SD). Qualitative variables were expressed as percentages. 95% confidence intervals (95% CI) for sample proportions were calculated using Wilson’s method. Statistical analyses were performed using STATA1 software, version 12 for Windows. Analysis was restricted to the *P. falciparum* group.

## Results

### Four cases of PMNS after imported malaria (Table 1)

Four of 2314 patients treated in the hospital for imported malaria during the study period fit the case definition of PMNS. Therefore, the estimated PMNS incidence rate for the hospital was 1.7 per 1000 malaria cases overall (95% CI 0.7–4.0 per 1000).

**Table 1:** Main clinical characteristics for cases of post-malaria neurological syndrome following *P. falciparum* (A), *P. vivax* (B) and mixed (C) infections

	N	Age	Gender	Cerebral malaria	Severe malaria	Treatment of malaria	Symptom-free period (days)	Fever	Confusion	Seizures	Psychosis	Cerebellar involvement	Motor deficit
A—PMNS post *P. falciparum*													
Case 1	1	33	M	No	Yes	AD/AP	10	Yes	Yes	No	Yes	Yes	No
Case 2	1	29	M	No	No	AP/Q/AD	13	Yes	Yes	Yes	Yes	No	Yes
Case 3	1	36	F	No	No	AP	15	Yes	Yes	No	Yes	Yes	No
Case 4	1	43	F	No	Yes	Q/MF	15	No	No	No	No	Yes	No
Nguyen [4]	22	29^a^	15 M	Yes 13 No 9	Yes 21 No 1	Q46% MF77% O18% AD59%	1–60 (median 4)	Yes 9 No 13	Yes 15 No 7	Yes 8 No 14	Yes 6 No 16	Yes 1 No 21	Yes 0 No 22
O’Brien [6]	1	42	M	Yes	Yes	Q/D	45	No	Yes	No	No	No	No
Zambito [7]	1	60	M	Yes	Yes	Q/D	11	No	Yes	No	No	Yes	Yes
Mizuno [8]	1	54	M	Yes	Yes	AD/MF	21	Yes	Yes	No	No	No	No
Nayak [9]	1	23	M	Yes	Yes	AD/O	7	No	No	No	No	No	Yes
Prendki [10]	1	19	M	Yes	Yes	Q	46	Yes	Yes	Yes	Yes	No	No
Prendki [10]	1	19	M	Yes	Yes	Q	7	Yes	Yes	Yes	No	No	No
Falchook [11]	1	50	F	No	Yes	Q/D/AP	11	No	Yes	No	Yes	Yes	No
Matias [12]	1	61	M	No	Yes	Q/D	2	Yes	Yes	No	Yes	Yes	Yes
Markley [13]	1	42	M	Yes	Yes	MF/Q/D/AP	19	No	Yes	No	No	Yes	No
Forestier [14]	1	50	M	No	No	AP	21	No	Yes	No	No	No	No
Rakoto [15]	1	16	M	Yes	No	Q/AD/O	13	No	Yes	Yes	No	Yes	No
Pace [16]	1	48	F	No	No	AD/O	2	No	No	No	No	No	Yes
Caetano [17]	1	60	M	No	No	NA	NA	No	Yes	Yes	NA	Yes	No
Mohsen [18]	1	30	F	No	Yes	Q/D	35	No	Yes	Yes	No	No	No
Schnorf [19]	1	34	F	Yes	Yes	Q/O	17	Yes	No	Yes	No	Yes	No
Schnorf [19]	1	61	M	Yes	Yes	Q/MF	10	Yes	No	No	No	Yes	No
Agrawal [20]	1	12	F	Yes	Yes	Q/D	16	No	Yes	Yes	No	No	No
Rachita [21]	1	4	F	No	No	Q	7	Yes	No	No	No	No	Yes
Lawn [22]	1	44	M	No	Yes	Q/AF	7	Yes	Yes	No	No	No	No
Lawn [22]	1	22	F	No	Yes	Q/AF	3	Yes	Yes	No	No	Yes	No
Total	46		30 M	24	38	Q28, MF21, AD19, AF14, D7, O2		21	33	16	12	13	6
% [95% CI]			65.2 [50.7–77.3]	52.2 [38.1–65.9]	82.6 [69.3–90.9]	Q 60, MF 46, AD 41, AF 30, D 18, O 8		45.6 [32.1–59.8]	71.7 [57.4–82.7]	34.8 [22.7–49.2]	26.1 [15.6–40.3]	28.3 [17.3–42.6]	13.0 [6.1–25.7]
Mean (SD)		33.3 (12.7)					15.4^b^ (12.0)						
Median (min–max)		29 (4–61)					13^b^ (2–46)						
B—PMNS post *P. vivax*													
Goyal [23]	1	1.5	F	Yes	Yes	AD/Q	7	Yes	Yes	No	No	No	Yes
Sidhu [24]	1	8	F	Yes	Yes	AD	7	No	No	No	No	No	No
Kochar [25]	1	55	M	No	No	CQ	14	No	No	No	No	No	Yes
Kasundra [26]	1	14	F	No	NA	NA	14	No	No	No	No	Yes	Yes
C—PMNS post mixed infections (*P. falciparum*/*P. vivax*)													
Koibichi [27]	1	24	M	No	Yes	Q	26	Yes	Yes	No	No	No	No
Mani [28]	1	Adult	F	NA	NA	D/AD	NA	No	No	No	No	No	Yes

*M* male, *F* female, *D* days, *NA* not available, *Q* quinin, *AD* artemisinin derivatives, *MF* mefloquine, *AF* anti-folic, *O* other, *SD* standard deviation, *[95% CI]* 95% confidence interval

^a^Mean on 22 patients

^b^Calculated on 23 available figured data

Case 1 was a 33-year-old Caucasian male. He was a pilot and flew routes between France, Guinea and the Republic of the Congo. In September 2016 he presented with fever, headaches and vomiting, and thereafter received treatment in Paris for severe malaria (positive thick drop for *P. falciparum* with 5 parasites/2 μL, positive HRP2 antigen test) with hepatic impairment (SGOT/SGPT 92/105 U/L and hyperbilirubinaemia (93 µmol/L, normal range < 25 µmol/L) but no neurologic involvement or any other severity criteria. The treatment regimen included intravenous artesunate (2.4 mg/kg, 5 doses for 3 days) then atovaquone/proguanil (1000/400 mg per day for 3 days), and the patient improved quickly, both clinically and biologically (blood smear negative for *P. falciparum* on day 3). On day 7, he presented headaches and fever (38 °C) and on day 8 abdominal pain, nausea and vomiting. The renewed blood smear was negative. On day 10, the patient showed confusion, ataxia, tremor, and dysarthria, and his fever increased to 39 °C. On day 11, he was given ceftriaxone for presumed enteric fever. On day 12, he remained confused and started having visual hallucinations and urine incontinence. CSF analysis showed lymphocytic meningitis (Table 2), MRI was normal and EEG revealed asymmetric (right) frontal slowing. Laboratory results showed no inflammation, a slight hyperbilirubinaemia that diminished over the first days and a weak positive titre of anti-nuclear factor (1/80) with no positivity for anti-DNA. Thereafter, he was treated with cefotaxime and acyclovir from day 12–21 (until a second CSF analysis showed no viral or bacterial infection), and corticosteroids from day 15–30 (methylprednisolone 500 mg/od for 3 days then prednisone 1 mg/kg/od), with clinical improvement on day 19. The patient was discharged with only a slight residual cerebellar ataxia on day 29 and had fully recovered on day 60.

**Table 2:** Biological and radiological features of PMNS post *P*. *falciparum*, *P. vivax* or mixed infection

	CSF (WBC/%L)	CSF protein (g/L)	CRP (mg/L)	EEG	MRI	MRI matching ADEM or AIE
*P. falciparum* infection						
Case 1	32/90	1.05	N	Abnormal	N	–
Case 2	82/87	2.41	N	Abnormal	Limbic and hippocampal hypersignal	ADEM plausible
Case 3	173/89	1.88	40	Abnormal	N	–
Case 4	NA	NA	N	NA	NA	
Nguyen [4] (N = 22)	> 5 in 8/lymphocytic predominance	> 0.5 in 13	NA	NA	NA	
O’Brien [6]	NA	NA	NA	NA	WM lesions in CH, brainstem, cerebellum, thalamus and basal ganglia	ADEM plausible
Zambito [7]	20/100	0.86	NA	Abnormal	N	–
Mizuno [8]	10/100	0.83	27	Abnormal	N	–
Nayak [9]	N/N	0.66	NA	NA	NA	
Prendki [10]	76/100	0.52	163	Abnormal	N	–
Prendki [10]	26/91	1.88	9	Abnormal	N	–
Falchook [11]	NA	NA	N	NA	Pons, posterior internal capsule, thalamus, corona radiata, and periventricular hypersignal	ADEM unlikely
Matias [12]	N/N	1.83	N	Abnormal	Extensive demyelinating lesions (subcortical WM and cerebellum)	ADEM or dysimmune plausible
Markley [13]	20/100	0.92	NA	Abnormal	N	–
Forestier [14]	43/95	1.2	N	Abnormal	N	–
Rakoto.[15]	31/98	2	N	NA	NA	
Pace [16]	NA	NA	8	NA	Brainstem and spinal cord high signal and swelling	ADEM plausible
Caetano [17]	123/100	1.88	NA	Normal	N	
Mohsen [18]	22/100	1.4	NA	Abnormal	Subcortical unilateral frontal and temporal, and cerebellar hypersignal with gadolinium enhancement	ADEM unlikely but not impossible
Schnorf [19]	10/95	0.6	NA	Abnormal	Peri and supraventricular and cerebellar hypersignal	ADEM plausible
Schnorf [19]	80/87	1.8	NA	Abnormal	N	
Agrawal [20]	5/100	1.12	NA	NA	Asymmetric supraventricular, semi-ovale center, genu of corpus callosum WM hypersignal	NA
Rachita [21]	7/100	1.25	NA	NA	Multifocal asymmetric diffuse WM hypersignal with small mass effect	ADEM
Lawn [22]	N/N	0.89	N	NA	N	–
Lawn [22]	59/100	2.89	N	Abnormal	N	–
Total abnormal	25/42	33/42	5/14	14/15	9/21	
% abnormal [95% CI]	59.5 [44.5–72.9]	78.6 [64.1–88.3]	35.7 [16.3–61.3]	93.3 [70.2–98.8]	42.8 [24.5–63.5]	
Mean WBC/%L (SD)	48^a^/96^a^ (46)/(5.1)	1.4^b^ (0.6)	49.4^c^ (64.9)			
Median WBC/%L (min–max)	31^a^/100^a^ (5–173)/(87–100)	1.2^b^ (0.5–2.9)	27^c^ (8–163)			
*P. vivax* infection						
Goyal [23**]**	70/NA	0.5	NA	NA	Diffuse periventricular, deep and subcortical WM hypersignal	
Sidhu [24]	NA	NA	NA	NA	Subcortical, cortical, left parietal periventricular regions and pons hypersignal	
Kochar [25]	NA	NA	NA	NA	NA	
Kasundra [26]	10/100	0.65	NA	NA	T1-weighted isointense and T2 and fluid-attenuated inversion recovery high signal in bilateral cerebellar hemispheres including vermis	
Mixed infection						
Koibuchi [27]	30/NA	0.46	52	NA	Asymmetric spotty mottled cortical and subcortical lesions	
Mani [28]	NA		NA	NA	Multifocal confluent areas of demyelination in the corpus callosum and periventricular region, myelitis	

Meningitis is defined in the CSF by CSF WBC ≥ 5/mL. CSF Protein ≥ 0.5 g/L is considered abnormal. CRP normal value ≤ 5 mg/L

*CSF* cerebrospinal fluid, *WBC* white blood count, *%L* proportion of lymphocytes, *CRP* c-reactive protein, *WM* white matter, *NA* not available, *N* normal, *ADEM* acute disseminated encephalomyelitis, *AIE* autoimmune encephalitis, *LP* lumbar puncture, *SD* standard deviation, *[95% CI]* 95% confidence interval

^a^Calculated on abnormal and available figured data, n = 17

^b^Calculated on abnormal and available figured data, n = 20

^c^Calculated on abnormal and available figured data, n = 5

**Fig. 1:**
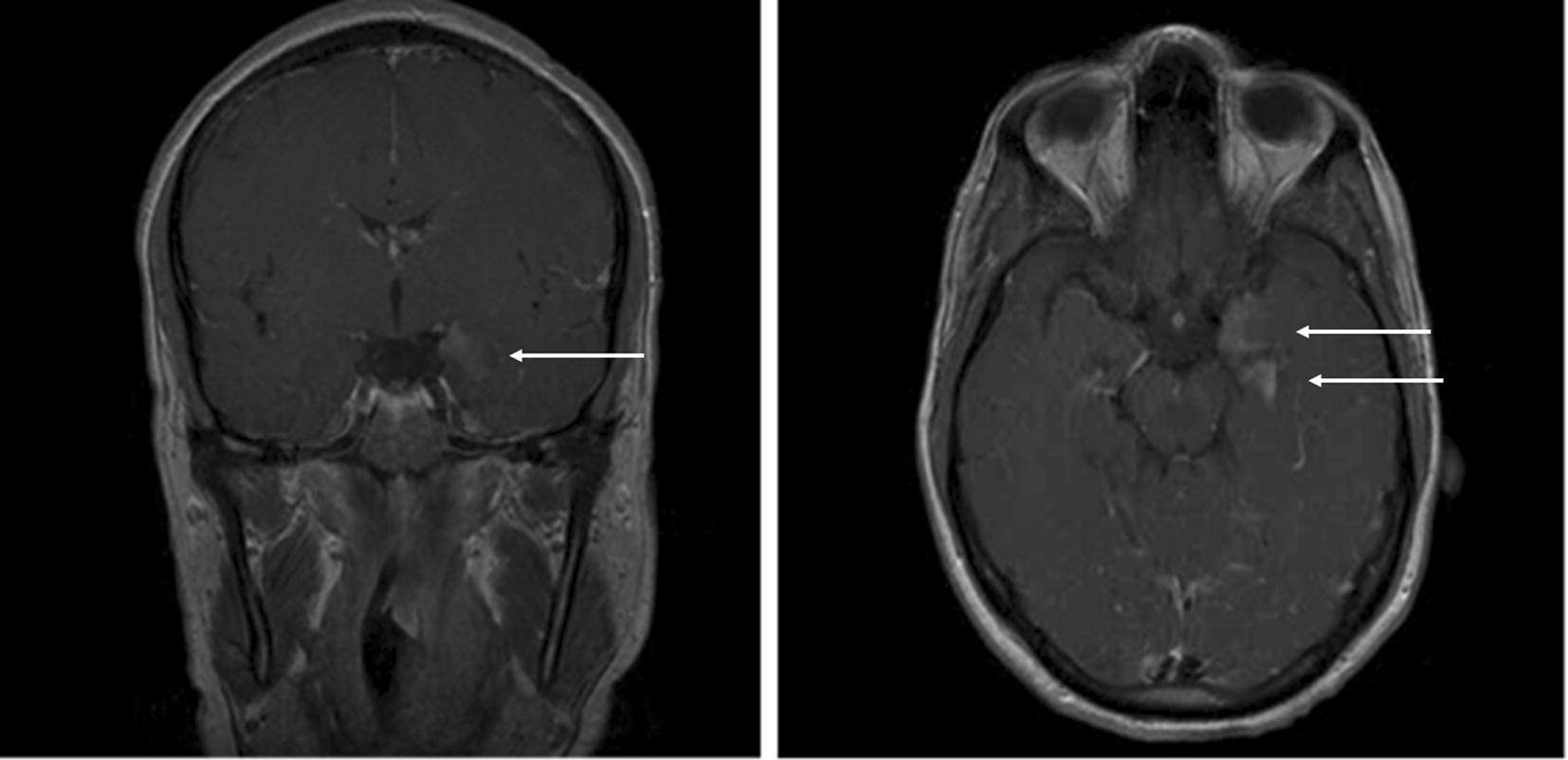
T1 gadolinium-enhanced MRI sequences at day 48 (case 2). Left limbic and hippocampal hypersignals are indicated with white arrows

Case 2 was a 29-year-old Caucasian male who worked in Ivory Coast. He presented a first episode of acute falciparum malaria without severity criteria in July 2013. That episode was treated with a 3-day course of atovaquone/proguanil (standard treatment). The patient experienced a second symptomatic episode with a positive blood smear (0.18%) 3 weeks later and received a 3-day course of artemether/lumefantrine with good outcome. He then consulted on day 37 for vomiting and a 40 °C fever for which intravenous quinine was initiated despite a negative blood smear. On day 38, he presented convulsions and a severe alteration of consciousness requiring sedation and ventilation. He was evacuated to Paris where his anti-malarial treatment was changed to artesunate despite a still negative blood smear. On day 42, sedation was stopped but the patient presented visual hallucinations and generalized convulsions and consequently had to be re-sedated. The blood test was again negative for malaria but an HRP2 antigen test was positive. T2 and T1 gadolinium-enhanced MRI sequences on day 48 showed hippocampal lesions (Fig. 1), EEG diffuse slowing, and CSF analysis lymphocytic meningitis (Table 2a). Differential diagnoses such as infectious or inflammatory/immunological diseases were ruled out (tests for all of the following were negative or normal: HSV, EBV, VZV, HIV, VHB, VHC, HHV6, adenovirus, dengue fever, Chikungunya, Rift Valley fever virus, West-Nile virus, *Borrelia*, *Coxiella*, *Brucella*, *Bartonella*, *Tropheryma whipplei*, gram-negative bacilli, TPHA-VDRL, African trypanosomiasis, cysticercosis, toxocariasis, anti-nuclear factor (ANF), anti-neutrophil cytoplasmic antibodies (ANCA), complement, anti-phospholipid (APL) antibodies, anti-neuronal antibodies including NMDA-receptor in CSF, rheumatoid factor, anti-CCP, angiotensin conversion enzyme and 16S RNA on CSF). The patient improved around day 54 without specific treatment. MRI on day 63 had returned to normal. On day 73, the patient was free of neurological symptoms.

Case 3 was a 36-year-old Caucasian female who lived in France but had visited friends in Mali in November 2009. On 17 November, she complained of fever, diarrhoea and vomiting and on the 18th returned to Paris. She was diagnosed with non-severe (2% parasitaemia) falciparum malaria on 28 November and treated with a 3-day course of atovaquone/proguanil with good outcome. Thirteen days later (day 15) she became confused, aphasic and anosognosic. CSF analysis showed lymphocytic meningitis (Table 2). EEG showed frontal bilateral slowing and MRI no abnormalities. Infectious investigations were all negative (including HSV, VZV and enterovirus, HIV, Chikungunya, dengue, West Nile and Rift Valley fevers, yellow fever and African trypanosomiasis). Intravenous acyclovir was stopped after 2 days following the negative result for HSV PCR in CSF. On day 19, she was discharged with partial recovery. On day 22, she was re-admitted for confusion, aphasia, ataxia, delirium, and fever (38 °C). MRI remained unremarkable but EEG showed major frontal slowing and spike-and-wave discharges. Treatment with acyclovir and levetiracetam was re-initiated for 3 weeks. The patient’s clinical status gradually improved allowing for her discharge on day 41. She had fully recovered on day 87.

Case 4 was a 43-year-old Caucasian female who presented with severe falciparum malaria (4.5% parasitaemia) in July 1999 after a trip to Ivory Coast. She was initially treated with intravenous quinine (25 mg/kg/day) and on day 5 was given an oral dose of quinine (500 mg tid) after which she developed hypoacusis. On day 6, she was treated with mefloquine (6 × 250 mg for 24 h). She was discharged after recovery but on day 18 she experienced dizziness, limb weakness, gait impairment, nausea, an increase in pre-existing headaches and an episode where she was unable to read. Tremor and ataxia were observed during a resulting physical examination. On day 21, the level of mefloquine in her blood was high (5 µg/L, HPLC method, normal value < 1.5 µg/L). On day 27, her clinical status improved spontaneously and she was discharged without any specific treatment. Follow-up blood examinations showed that the half-life of mefloquine elimination for this patient was 9 days. On day 41, the patient had a normal clinical status and no *sequelae*.

### Literature review (Table 1)

The systematic review of the literature found 48 PMNS patient cases of which 42 involved *P. falciparum* alone, 4 *P. vivax* alone, and 2 *P. falciparum* and *P. vivax* together. Of these 48 patients, 16 were travellers (15 *P. falciparum* and 1 *P. vivax)* who acquired malaria while abroad and 32 were inhabitants of countries endemic for the infection (27 *P. falciparum* and 5 *P. vivax*). No cases were described with *P. ovale*, *P. malariae* or *P. knowlesi*.

### Matching the results of the local cases and those of the literature

The main clinical findings are summarized in Table 1. Four patients presented PMNS after *P. vivax* infection alone [23–26] and 2 after mixed malaria infections [27, 28]. Three were children, all after *P. vivax* infection [23, 24, 26]. One PMNS patient presented bilateral facial palsy that recovered spontaneously [25]. Table 2 summarizes CSF, CRP, EEG and MRI findings. In the *P. falciparum* group, of the 42 CSF analyses available, 25 (60%) showed lymphocytic meningitis (mean of 48 cells/mm^3^), and 33 (78%) high protein levels (mean of 1.4 g/L) without low glucose levels. When abnormal, EEG showed diffuse or frontal slowing. Computed tomography was never abnormal when performed (9/9). MRI revealed brain abnormalities in 9 of 21 *P. falciparum* patients (43%), showing hypersignals, sometimes with gadolinium enhancement, in different localizations (periventricular, sub-cortical, corpus callosum, brainstem, cerebellar), usually with little extension. One child showed clinical and MRI characteristics of severe ADEM [20]. One case presented extensive myelitis. Four and two patients with *P. falciparum* and *P. vivax* infection, respectively, showed grey matter hypersignals.

### Treatment and outcome

In the *P. falciparum* group, corticosteroids were used to treat 10 patients, among whom 6 had MRI abnormalities. Six of these 10 patients received intravenous high-dose methylprednisolone (with oral tapering). Of the 4 patients in the *P. vivax* group, 3 received corticosteroids (missing data for one) and one in the mixed infection group. None of the 22 patients with follow-up data had *sequelae*. The mean time to recovery in the 21 patients with that datum was 17.4 days (12.3), (min–max, 2–45 days). The characteristics of PMNS are summarized in Table 3.

**Table 3:** Summary of PMNS due to *Plasmodium falciparum*

*Plasmodium falciparum* PMNS features (determined on 46 published cases)	
Mean age	33 years
Sex ratio (% male)	66%
Previous severe malaria	85%
Previous cerebral malaria	50%
Malaria species	*Plasmodium falciparum* (also *P. vivax*^a^)
Previous treatment for malaria	No effect
Traveler or local population	Described in both populations
Symptom-free period since malaria (mean)	15 days
Fever	46%
Mental confusion	72%
Seizures	35%
Psychosis	26%
Cerebellar disorders	28%
Motor deficit	13%
MRI (abnormal)	43% mostly white matter lesions
CSF	52% lymphocytic meningitis, 77% high protein level but normal glucose level
EEG (abnormal)	94% (encephalopathy)
Treatment	Corticosteroids advised in severe forms (lack of specific recommendation)
Prognosis	Excellent (100% *ad integrum* recovery)

^a^See text for further details

## Discussion

For the present work, the literature’s 48 cases of PMNS published since 1997 were assessed and 4 local imported cases added thereto. From the database of the hospital, an incidence of 1.7 per 1000 was determined, comparable with that of 1.2 per 1000 reported in a previous study [4].

In the studied patient population, PMNS developed mostly in adults (mean age of 33) after severe falciparum malaria (85%), with cerebral malaria in 50% of the cases. It was seen most frequently in people who live in (two-thirds of reported cases), but also in those who travel to, malaria-endemic areas. PMNS was also seen after vivax malaria, notably in children in 50% of the cases [23, 24, 26]. There was a mean symptom-free period of 15 days before the onset of PMNS symptoms. The most common clinical feature was confusion, followed by fever, seizures, psychosis, cerebellar involvement, and motor deficits. More rarely, cranial nerve palsy [25], visual impairment [26, 27], sphincter disorders [16], and headaches were seen. When performed, CSF analyses were pathological in 60% of cases, showing high protein levels (1.4 g/L), and lymphocytic meningitis (96% lymphocytes). White matter abnormalities were always present in abnormal (43% of the cases) MRI studies. However, clinical cerebellar impairment did not appear to be associated with the presence of cerebellar lesions in MRI (only 2 patients with cerebellar hypersignals). MRI studies in post-*P. vivax* PMNS, appeared to show the same types of lesions (noting however that there were only 3 available [23, 24, 26]). EEGs, when realized, usually showed signs of encephalopathy.

A study from Vietnam had concluded that mefloquine was a risk factor for PMNS after severe malaria (relative risk of 7.4; 95% CI 2.5–22) [4]. However, only 3 of the 19 patients published after that study, and only one of the local patients, had been treated with mefloquine. Also, half of the *P. falciparum* patients reported had received a range of treatments (including artemisinin combination therapy). Moreover, PMNS occurred after *P. vivax* infection for which mefloquine had not used. Considered together, these observations cast doubt on an association or causative effect for mefloquine and PMNS.

Cerebellar signs can be seen not only in severe malaria, where they respond to anti-malarial treatment [29–31], but also in PMNS, where the inefficacy of anti-malarials and the absence of parasites argue for a different, perhaps immune, mechanism. Overall, 30% of the studied PMNS cases presented cerebellar involvement and various neurological signs and symptoms (Table 1). Therefore, PMNS should include DCA as they share the same prognosis and outcome [4, 19].

PMNS shares many features with CNS post-infectious diseases such as ADEM and auto-immune encephalitis. ADEM [32–37] is a rare, acute, post-infectious (bacterial or viral) or post-vaccinal syndrome. It is a monophasic disease characterized by multiple inflammatory demyelination lesions. Its signs classically appear 2–30 days after the infectious trigger. The clinical picture may include fever, headaches, encephalopathy, seizures, sensorimotor focal deficits, acute cerebellar ataxia, cranial nerve palsy, myelitis, and optic neuritis. CSF analyses only show lymphocytic pleiocytosis and high protein content. CT scans are pathological in up to 30% of cases. MRI show diffuse, asymmetric signs of white matter demyelination and more rarely grey matter involvement in the cortex, the thalamus, basal ganglia [32, 37–39] and the spinal cord. With corticosteroid treatment, the outcome is usually good and free of *sequelae* [32]. Multiple pathophysiological hypotheses have been proposed, including immunization against some cerebral antigens after a neurotropic infection, mediated by molecular mimicry and T cell-activated cerebral aggression [35, 40]. Although *P. falciparum* and *P. vivax* have not been identified as causative agents for ADEM, the great number of characteristics shared between this latter and PMNS suggest that the relationship is nonetheless highly plausible.

In 2007, a new group of neurological disorders mediated by neuronal antibodies (against ions channels and synapses) appeared in the literature under the term auto-immune encephalitis (AIE) [41]. AIE causes neurological and psychiatric impairment usually in a setting of malignancy or post-viral infection (herpes virus, for example) [42]. Various antibodies have been described, some leading to encephalitis or cerebellar ataxia, i.e., *N*-methyl-d-aspartate-receptor antibody-NMDAR or P/Q type voltage-gated-calcium-channel antibody. The NMDAR antibody was described in 12 (24.5%) of 49 patients 3 months after HSV encephalitis [43]. No neuronal auto-antibodies have been found to be related with malaria but new auto-antigens are being discovered at a pace of about one per year [35]. In AIE as in PMNS, MRIs are normal in about 50% of cases, improvement is observed after corticosteroids and *sequelae* are largely absent.

The clinical features of PMNS are compatible with post-infectious encephalitis, either ADEM or AIE. Symptom-free latency and negative extensive screening of possible infectious or systemic causes argue for a post-infectious immunologically mediated cerebral aggression. CSF analyses, inflammation characteristics and EEG are all compatible with post-infectious encephalitis (Table 2). For ADEM, MRI is a key point for diagnosis as it is almost always abnormal, to the point where a normal MRI is a commonly-accepted criteria against it [35, 44]. It is important to note however that an MRI may be normal in ADEM when performed too early in the course of the disease [38, 45]. In AIE, MRI shows no abnormalities in 50% of cases and non-specific abnormalities in the other 50%. In PMNS, less than half of MRIs are pathological. Therefore, cases of PMNS with normal MRIs, could involve AIE.

**Fig. 2:**
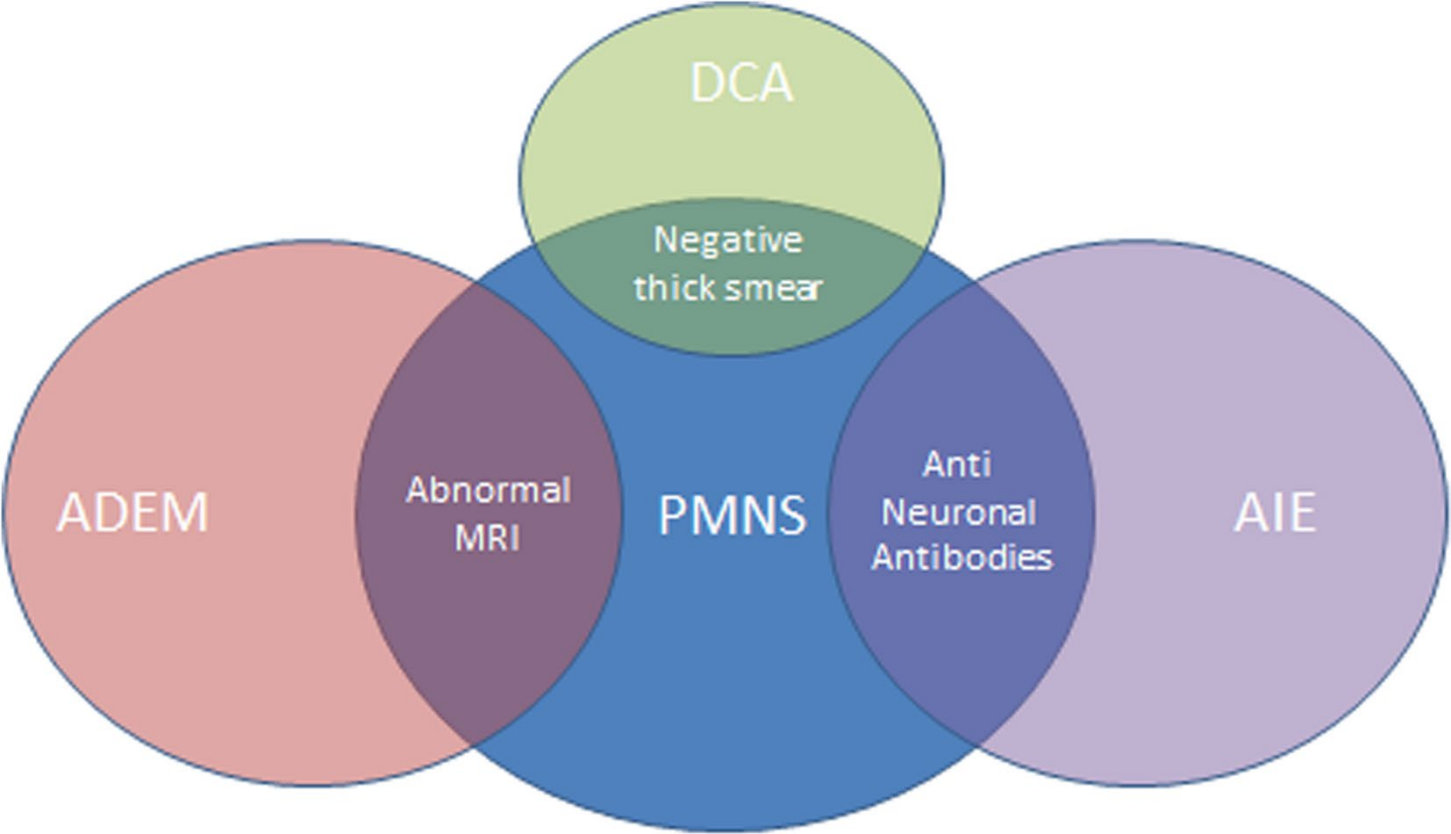
Nosological framework for post-malaria neurological syndrome. DCA is difficult to classify in PMNS because in most of the published cases, data on parasite clearance was lacking. However, other elements (delay, lack of sensitivity to antimalarials) advocate for at least a relationship between them. Delayed post-infectious cerebellar involvement was described in a number of the confirmed PMNS cases; MRIs were normal for some and ADEM-compatible for others. ADEM: acute diffuse encephalomyelitis. *MRI* magnetic resonance imaging, *DCA* diffuse cerebellar ataxia, *AIE* autoimmune encephalitis, *PMNS* post-malaria neurological syndrome

The mechanisms underlying PMNS are poorly understood. The delayed onset, negativity of blood smear, association with fever, and sometimes elevated CRP may suggest an inflammatory pathway, probably involving cross presentation like in AIE, but no demonstration of that has been made to date. The authors of the only study on inflammatory pathways of malarial post infectious neurological complications reported increased blood and CSF pro-inflammatory cytokines, like TNF, IL-6 and IL-2, in 12 patients with DCA (compared to 8 patients without it) [46]. PMNS has not been investigated in that manner. The approximately 15-day symptom-free period is not without interest, since it corresponds roughly to the 2–3 weeks needed for the production of specific antibodies. The cerebral microvasculature could be the locus of this immunization process, since parasites and pigments are known to be sequestered there due to cytoadherence; this is the case even after *P. falciparum* clearance and even in non-neurological malaria [47]. The fact that cytoadherence is less significant, or at least less frequent, [48, 49] in *P. vivax* infection could explain why most PMNS cases follow *P. falciparum* infections. The first case presented non-specific anti-nuclear factors and the second had neither anti-nuclear factors nor anti-neuronal antibodies. Recently, Sahuguet et al. [50] reported a case of AIE with anti-voltage-gated-potassium-channel antibodies in the setting of PMNS, and in so doing opened a field of pathophysiological possibilities and made autoimmune investigations useful. The cases of PMNS with normal MRIs should accordingly be investigated as AIE. It appears that PMNS is a post-infectious syndrome probably due to various pathophysiological mechanisms (Fig. 2).

The use of steroids in PMNS is another topic worth discussing. According to the follow-up data available, all patients fully recovered in a mean delay of 17.2 days (2–45 days; delay to recovery could not be determined for post-*P. vivax* patients) and most without steroid treatment. However, steroids were given to the 14 most-severe cases (including 11 with MRI abnormalities). This observation casts a shadow on the ‘self-limiting course’ [13] of PMNS, forwarded by most of the reports in the literature, and raises questions as to whether or not steroids contribute to the cure of severe cases, reduce *sequelae* or shorten time to recovery. An interventional study would be needed to respond to these questions. In analogy with AIE treatment, corticosteroids do nonetheless appear to be a first-line treatment for PMNS, at least in severe cases and at least for now. The prognosis of PMNS seems to be good and corticosteroid treatment should be discussed for the most severe cases. If corticosteroids are not effective, first the diagnosis of PMNS should be reconsidered, and thereafter intravenous immunoglobulin or plasma exchange discussed.

## Conclusion

PMNS is a rare entity englobing a range of neurological disorders following malaria cure. There appears to be no direct role for the parasite but rather a putative immune-mediated aggression of the CNS. Most of its characteristics can fit into the diagnosis of post-infectious encephalitis, ADEM or AIE. The addition of malaria due to *P. falciparum* and *P. vivax* parasites to the list of pathogens causing ADEM should be highly considered. Further studies on this rare syndrome should include immune investigations (i.e., auto-antibodies).
